# Supplementary material for: Added value of clinical prediction rules for bacteremia in hemodialysis patients: An external validation study
Source: PLoS One. 2021 Feb 22;16(2):e0247624. doi: 10.1371/journal.pone.0247624 (PMC7899347; doi:10.1371/journal.pone.0247624)
Supplement: S1 Table — (DOCX) [file pone.0247624.s001.docx]

**S1 Table.** Pathogens involved in bacteremia in validation cohorts 1 and 2

|  | Validation cohort 1  *n* = 37 | Validation cohort 2  *n* = 16 | Total  *n* = 53 |
| --- | --- | --- | --- |
| *Staphylococcus aureus (S. aureus)* | 9 (24.4) | 9 (56.2) | 18 (34.0) |
| [methicillin‐resistant *S. aureus*] | 5 | 2 | 7 |
| coagulase negative S*taphylococci* | 1 (2.7) | 2 (12.5) | 3 (5.7) |
| Streptococcus spp. | 2 (5.4) | 2 (12.5) | 4 (7.5) |
| Enterococcus spp. | 2 (5.4) | 2 (12.5) | 4 (7.5) |
| *Escherichia coli* | 11 (29.7) | 0 | 11 (20.8) |
| *Klebsiella pneumoniae* | 4 (10.8) | 1 (6.3) | 5 (9.4) |
| Others | 8 (21.6) | 0 | 8 (15.1) |
